# Formulation and Evaluation of Niosomal Alendronate Sodium Encapsulated in Polymeric Microneedles: In Vitro Studies, Stability Study and Cytotoxicity Study

**DOI:** 10.3390/nano12203570

**Published:** 2022-10-12

**Authors:** Ahlam Zaid Alkilani, Hana Abu-Zour, Anas Alshishani, Rana Abu-Huwaij, Haneen A. Basheer, Hadeel Abo-Zour

**Affiliations:** 1 Department of Pharmacy, Faculty of Pharmacy, Zarqa University, Zarqa 13110, Jordan; 2 Faculty of Pharmacy, Amman Arab University, Amman 11953, Jordan

**Keywords:** alendronate sodium, transdermal, niosomes, microneedles, permeability, BCS class III

## Abstract

The aim of this study is to design and evaluate a transdermal delivery system for alendronate sodium (ALS) loaded with nanocarrier to improve its permeability and prolong its release. This is due to its low bioavailability, potential gastrointestinal side effects, and the special administration needed for the oral dosage form of ALS. When using the ether injection method, various niosomal formulations were produced. Size of the particles, polydispersity index (PDI), surface charge (ZP), drug entrapment efficiency (EE), and in vitro release were used to characterize the resulting niosomes. The size of niosomes ranged between 99.6 ± 0.9 and 464.3 ± 67.6 nm, and ZP was from −27.6 to −42.27 mV. The niosomal formulation was then loaded to aqueous polymer solution of 30% polyvinyl pyrrolidone (PVP) (MN-1), 30% PVP with 15% poly(vinyl alcohol) (PVA) (2:1) (MN-2), and 30% PVP with 15% PVA (1:1) (MN-3). The cumulative amount of ALS (Q) was in the following order: MN-1 > MN-2 > MN-3. All formulations in this study were stable at room temperature over two months, in terms of moisture content and drug content. In conclusion, a transdermal delivery of ALS niosomes combined in microneedles (MNs) was successfully prepared to provide sustained release of ALS.

## 1. Introduction

The second most significant global health issue, following cardiovascular disease, is osteoporosis [1]. It is a bone disease that worsens with time and is defined by a loss of bone mass and density, which can increase the risk of fracture [2]. According to studies, one in five men and one in three women over the age of 50 in the world experience osteoporotic fractures [1]. Alendronate sodium (ALS) is one of drugs generally considered the first option for the treatment of osteoporosis, due to the evidence of its “broad spectrum” anti-fracture efficacy [3]. The BCS class III classification of ALS indicates that the drug is readily soluble but has a limited permeability [4]. 

There are many issues associated with the use of ALS, including the extreme low bioavailability of approximately 0.9–1.8%, esophageal ulcers, and complicated administration which leads to poor patient compliance [3,5]. In order to prevent esophageal irritation and esophageal cancer, it is advised that ALS be provided following a prolonged fast, with a full glass of water, while standing up for at least 30 min [3]. Therefore, the development of an alternative route of administration which overcomes the drawbacks of the orally administered alendronate is needed [3]. 

Transdermal drug delivery (TDD) has grown in popularity as a non-invasive delivery method that is simple to administer to more vulnerable age ranges while avoiding some of the bioavailability issues that arise with oral drug delivery because of limited absorbability and metabolic concerns [6]. However, due to the structure of the stratum corneum (SC) and its hydrophobic properties, not all drugs are suitable for transdermal administration [7]. To address this issue, numerous unique TDD methodologies have undergone considerable development and have become attractive administrative techniques [8]. Passive technologies involve the use of chemical penetration enhancers, prodrug, eutectic systems, and nanotechnology [9]. In addition, physical methods can be also used to enhance the permeation of drugs, such as microneedles (MNs). Due to their adaptability and capacity for sustained release, the integration of MNs with nano-systems has become more popular. Therefore, integrating physical and chemical technology provides a significant improvement in drug delivery. Niosomes as a passive method will permit sustained drug release over a prolonged period of time. The use of MNs in combination with niosomes is the best possible approach to enhance the permeability and sustain the release of BCS class III drugs.

In this study, we aim to develop and evaluate an integrated system consisting of nanomedicine combined with MNs for the transdermal delivery of ALS to improve its permeability and sustain its release. Therefore, an alternative formulation for the oral administration of ALS was studied in an effort to reduce GI side effects and enhance patients’ compliance. A novel dissolving microneedle (DMN) containing ALS niosomes was fabricated and evaluated by micromolding technologies using different biodegradable polymers.

## 2. Materials and Methods

### 2.1. Materials

Alendronate sodium was obtained as a gift sample from the JOSWE pharmaceutical company (Amman, Jordan). Phosphoric acid was purchased from the BBC chemicals laboratory, while HPLC grade acetonitrile and HPLC grade methanol were purchased from Tedia™, (Fairfield, OH, USA). Span™ 60, Tween™ 60, Tween™ 80, and cholesterol (Chol) were purchased from Sigma Aldrich™ (Dorset, UK). Diethyl ether and methanol were purchased from Tedia™, (Fairfield, OH, USA). A phosphate-buffered saline tablet was purchased from Sigma Aldrich™ (Dorset, UK). Cellulose dialysis membranes with a molecular weight cut-off (MWCO) of 12–14 kDa, average flat width of 28.46 mm, and average diameter of 17.5 mm, were purchased from Himedia Laboratories™ (Maharashtra, India). Polyethylene glycol 400 (PEG 400) and methylene blue were purchased from GCC Diagnostics™ (Flintshire, UK). Dihexadecyl phosphate, polyvinyl pyrrolidone (PVP) extra pure (molecular weight 40,000), poly (vinyl alcohol) (PVA) molecular weight (approx. 145,000), phosphate buffer saline (PBS), and dicetyl phosphate (DCP) were obtained from Sigma Aldrich™ (Dorset, UK). All other chemicals used in this study were of analytical grade.

### 2.2. Methods

#### Preparation of Niosomes

Different niosomal formulations were prepared by an ether injection method using nonionic surfactants (Span 60, Tween 60, and Tween 80) and Chol at different concentrations, as shown in Table 1. In a brief, Chol and nonionic surfactants were dissolved in 8 mL of diethyl ether before being combined with 2 mL of methanol. The resultant solution was then slowly injected into 10 mL of PBS containing ALS using a microsyringe at a rate of 1 mL/min. The solution was continuously stirred using a magnetic stirrer at a temperature 60–65 °C. The slow injection of the lipid solution into the aqueous phase caused a quick vaporization of the ether due to the temperature differences between the two phases, which led to spontaneous vesiculation and the production of niosomes. All niosome dispersions were kept at 4 °C in the refrigerator. As a control, blank niosomes were produced under the same methods but without the inclusion of ALS.

### 2.3. Characterization of Niosomes

#### 2.3.1. Transmission Electron Microscope (TEM)

A transmission electron microscope (TEM, FEI Morgani 268, operating voltage of 60 kV, Eindhoven, Netherlands) and Mega View II digital camera were used to investigate the morphology of the niosomes. Before imaging, niosomes were spread out over a copper grid that had been coated with carbon and diluted with distilled water (1:2 *v*/*v*). Image J was used to evaluate the niosomes’ morphology.

#### 2.3.2. Particle Size (PS) and Zeta Potential (ZP)

Using a particle Zetasizer analyzer, the PS and PDI of the ALS-loaded niosomes were measured (Brookhaven 90 plus, Holtsville, NY, USA). The electrophoretic light scattering (ELS) method was used to determine the particles’ surface charges (ZP). The inbuilt Zetasizer software automatically displayed the PDI for the entire spectrum of particles analyzed. The findings of each experiment were expressed as mean SD, and each experiment was carried out in triplicate.

#### 2.3.3. Determination of Entrapment Efficiency 

Ultracentrifugation was used to extract the unentrapped drug from the niosomes. Briefly, 1.5 mL of each ALS–niosome suspension were ultracentrifuged for 1 h at 16,000 rpm at 4 °C using a Beckman Optima LE-80 K Ultracentrifuge (Beckman Coulter Inc., Fullerton, CA, USA). The unentrapped drug was extracted from the supernatant, and the niosomes were then ultracentrifuged while being washed three times with PBS.

The content of the entrapped drug was determined after dissolving 0.4 mL of the niosomes in 2 mL of isopropanol until they were clear and then diluted up to 10 mL with PBS. The samples were then sonicated for 5 min at room temperature (RT). The amount of entrapped ALS was measured using high-performance liquid chromatography (HPLC). The EE% was determined using Equation (1), as follows:

(1) $$\mathrm{EE}\left(\%\right)=\frac{\mathrm{Amount}\;\mathrm{of}\;\mathrm{entrapped}\;\mathrm{drug}}{\mathrm{Total}\;\mathrm{ALS}\;\mathrm{amount}}\times 100\%$$

where the total ALS quantity refers to the entire amount of ALS utilized during preparation, and the amount of drug entrapped is the actual amount of drug successfully encapsulated in the vesicles.

#### 2.3.4. Attenuated Total Reflectance—Fourier Transform Infrared Spectroscopy (ATR-FTIR)

A Perkin Elmer UATR-II was used to perform ATR–FTIR analysis. In absorbance mode, spectra were obtained with a resolution of 2 cm^−1^ and 32 scans per sample. The spectral data was exported in CSV format, and Spectrograph Version 1.2.15 was used for analysis. 

#### 2.3.5. Short Term Stability Study of ALS-Niosomes

A short-term stability study for the optimized formulations was investigated in terms of color, PS, PDIs, and EE. Based on the results of in vitro characterization, the optimum niosomal formulations were kept at 4 °C in glass vials for two months, and their color, PS, PDIs, ZP, and EE% were then examined after one and two months.

#### 2.3.6. In Vitro Drug Release Study

The in vitro release of the ALS–niosome suspension (previously separated from the unentrapped drug by ultracentrifugation) was carried out under sink conditions and a heating circulator set to 37 °C. A cellulose dialysis membrane with MWCO 12–14 kDa was washed and soaked in PBS. The receiver compartment (20 mL) was filled with PBS containing 20% (*v*/*v*) isopropanol which was added to maintain sink conditions. The apparatus was then properly sealed before 1.5 mL of ALS-loaded niosomes were introduced to the donor compartment on a pre-soaked membrane. A magnetic stirrer was used to continuously mix the receiver medium. Aliquots (1 mL) were withdrawn from the receiver compartment at certain time intervals (1, 2, 3, 4, 5, 6, 24, and 48 h) and replaced with the fresh medium. The amount of released ALS was determined by HPLC. All samples were stored at −20 °C until analysis. This experiment was performed in triplicate.

#### 2.3.7. Ex Vivo Study

Franz diffusion cells were used for the ex vivo experiments, which were conducted at 37 °C. Full-thickness skin from a rat’s back was used. The excised skin was cleaned with water, divided into pieces of the proper size, and frozen at 20 °C after the subcutaneous tissue was removed. Utilizing a diffusion cell device (PremeGear, Hellertown, PA, USA) with an aperture diameter of 15 mm and a diffusion surface area of 1.76 cm^2^, the permeation of ALS from niosomal formulation was assessed. The receiving phase, which contained PBS with 20% isopropanol, had a volume of 12 mL. With the SC surface in contact with the donor phase, the rat skin was prepared and placed between the donor and receptor compartments. Then, 1 mL of the optimized niosome formulations were placed in the donor compartment, and 1 mL of the sample was collected at 1, 2, 3, 4, 6, 24, 48, and 60 h from the receptor cell. The same volume of fresh solvent was used to replace the sample after each collection. The cumulative amount of ALS that permeated the membrane over time (Q) was examined versus time (t). The linear slope of the cumulative amount of ALS penetrated per unit area (Q/A) vs. time plot’s was used to compute the steady-state flux (Jss, g/cm^2^/h) [10]. Apparent permeability (P) was calculated according to Equation (2), as follows:

(2) $$P\;=\frac{\mathrm{Jss}}{\mathrm{Co}}$$

where Co is the amount of drug in the donor solution. Under sink conditions, it is assumed that the drug concentration in the donor compartment is significantly higher than that in the receptor compartment [11]. 

#### 2.3.8. Cytotoxicity Study

The MTT assay was used to determine the cytotoxicity of the niosomal formulation F4 and blank F4. Here, RPMI medium with 10% fetal bovine serum was used to maintain the (MCF-7) cell line at 37 °C in a humidified environment with 5% carbon dioxide (CO_2_). A 96-well plate with 5000 MCF-7 cells per well was seeded with the cells. To allow for cell adhesion and recovery, the cells had a 24 h incubation period. On the following day, 20 μL of different serial dilutions (0.92, 0.092, 0.0092, and 0.00092 mM) of ALS, as well as serial dilutions of F4 blank formula (1.52, 0.152, 0.0152, and 0.00152 mg/mL), were added to the appropriate wells and incubated for 72 h at 37 °C. After treatment, each well received 20 µL of a 5 mg/mL solution of the 4,5-dimethylthiazol-2-yl)-2,5-diphenyltetrazolium bromide (MTT) tetrazolium substrate, which was then incubated for 4 h at 37 °C. Following the removal of the medium, DMSO was used to dissolve the violet formazan crystals that had formed. By examining the plates on a microplate reader (Glomax™, Madison, WI, USA) at 450 and 570 nm, the samples’ absorbance was determined. Excel spreadsheets were used to plot cell survival (%) versus drug concentration (mM). For each cell line, each condition was repeated four times in three independent experiments. A graph showing cell survival (%) versus drug concentration was produced using Excel spreadsheets. The *T*-test was used to determine the statistical significance, and *p*-values were calculated, with *p* < 0.01 being considered significant.

#### 2.3.9. Fabrication of ALS Niosomes-Loaded MNs

**Figure 1:**
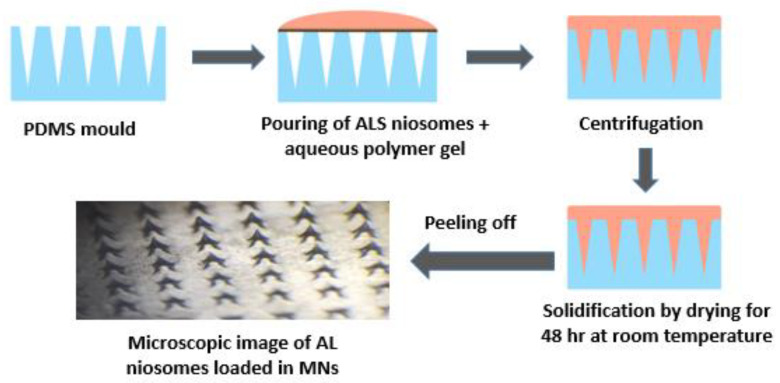
Schematic representation of the fabrication of ALS-loaded niosomes in MNs.

A range of biocompatible polymers were used in varied amounts to produce aqueous gels in order to fabricate polymeric MNs. Centrifugation assisted micromolding was used to manufacture MNs, which were then dried as previously described [12]. Briefly, 2.5 mL of polymeric solution and 1.5 mL of niosomes containing 2.44 mg of ALS were combined, as shown in Table 2. Prior to MN casting, the dispersion was carefully mixed for a few minutes to ensure the uniformity of the niosomes. A mixture of ALS-loaded niosomes and aqueous polymer solution weighing about 150 mg was poured into silicone molds (with pyramidal needles with dimensions of 600 mm in height, 300 mm in width, and 300 mm in interspacing), centrifuged at 2000 rpm for 20 min, and then dried for 48 h at RT (Figure 1). The MNs were then removed from the molds and visually examined for homogeneity and needle formation.

### 2.4. Characterization of Dissolving MN Arrays Loaded with ALS-Niosomes

#### 2.4.1. The Dissolution Rate of MN Arrays 

The hair on rat skin was shaved before the experiment to study the dissolution of the MNs after insertion, and methylene blue was loaded in the MNs for ease of observation. To prevent the skin from drying out, full thickness rat skin was placed, dermal side down, on a piece of tissue paper wetted with PBS, and MNs loaded with methylene blue were manually applied to the skin [13]. At specified intervals of 0, 5, 10, and 20 min, MNs were taken out of the skin and examined under an optical microscope to determine their dissolved morphology.

#### 2.4.2. Microneedles Insertion Studies

A commercial polymeric film (Parafilm™, Vernon Hills, IL, USA) was evaluated as a model membrane for MN insertion studies [14]. The Parafilm™ sheet was folded into an eight-layer film (1 mm thickness). A thumb was used to press the ALS-loaded niosomes in the MNs onto the Parafilm^TM^ for one minute at the MNs’ baseplate. Following insertion, the MNs were taken out of the Parafilm^TM^ sheet. Each layer of the Parafilm^TM^ sheet was opened up, and the number of holes was counted.

#### 2.4.3. Drug Content 

Drug content was measured by dissolving ALS-loaded niosomes in MNs in a 10 mL volumetric flask containing an isopropanol–PBS (30:70) mixture in a sonicator for 1 h at 37 °C, and then collecting 1 mL into 1.5 mL tubes and diluting with diluent up to 5 mL. This solution was centrifuged at 12,000 rpm for 10 min, and the supernatant was collected for quantification by HPLC. For content uniformity within an individual MN, drug recovery percentage was determined from different MNs. 

#### 2.4.4. Mechanical Characterization of ALS-Niosomes Loaded MNs 

Weights of 100 g, 200 g, 500 g, and 1000 g were placed on the tips of the MNs and held there for 5 min before being removed [15]. After that, the MNs were immediately viewed under a microscope to check on their morphology and fracturing.

#### 2.4.5. Short Term Stability Study of ALS-Niosomes Loaded MNs

For two months, the short-term stability of the ALS-loaded niosomes in polymeric MNs was assessed in terms of drug content and moisture content. The MN samples were accurately weighed and stored at room temperature in desiccators containing anhydrous calcium chloride. Samples were drawn out and weighed after one and two months. Equation (3) was used to calculate the moisture content percentage from the weight variations compared to the final weight [16], as follows:

(3) $$\%\;\mathrm{Moisture}\;\mathrm{content}\;=\left(\frac{\mathrm{Final}\;\mathrm{weight}-\mathrm{Initial}\;\mathrm{weight}}{\mathrm{Initial}\;\mathrm{weight}}\right)\times 100$$

#### 2.4.6. Ex Vivo Permeation Studies 

Ex vivo permeation studies were conducted using Franz diffusion cells. The volume of these cells is 12 mL, and the effective diffusion area is 1.76 cm^2^. To maintain sink conditions, the receptor chamber was filled with PBS containing 20% isopropanol (pH = 7.4) as a solubilizer. A magnetic stirrer was used to continuously stir the solution in the receiver compartments at 37 °C and, for one minute, MN-1, MN-2, and MN-3 were manually pressed into rat skin. The SC side was in touch with the donor phase, and the donor and receiver compartments were securely fastened with a clamp. The space between the two chambers was covered in waterproof film to avoid evaporation (Parafilm^TM^).

It was possible to measure the permeation of ALS through rat skin. By using a syringe to remove 1 mL aliquots from the receptor media at different time intervals (1, 2, 3, 4, 5, 6, 24, 48, and 60 h) and then instantly replacing the same volume with fresh PBS containing 20% isopropanol (pH = 7.4), the samples were then quantified by HPLC. 

The amount of ALS that penetrated through rat skin per unit surface area (Q/A) was plotted against time (t). The (Q/A) was calculated using the following Equation (4):

(4) $$Q\;=\left(\mathrm{Ci}\;V+\overset{n-1}{\underset{i\;=1}{\sum }}\left(C_{i}S\right)\right)∕A$$

where;

Q = the cumulative amount of drug permeated per surface area of membrane (μg/cm²);
Cn = the ALS concentration (μg/mL) determined at nth sampling interval;
V = the volume of the receiver solution in the Franz diffusion cell, namely 12 mL;
Σ Ci = the sum of concentration of ALS (μg/mL) calculated at sampling intervals 1 through n − 1;
S = the volume of the individual sample;
A = the surface area of Franz cell opening, namely 1.77 cm².

#### 2.4.7. Kinetic of Drug Release

Using LabPlot version 2.0, the drug release rates for niosome and MN formulations were fitted to the Korsmeyer–Peppas equation (Mt = K* × t^n^). The software was then given the task of determining the best fit line. Only fit curves with R^2^ ≥ 0.95 and the sum of squared residuals (SSD) ≤ sum of squares were considered appropriate.

#### 2.4.8. Analysis of ALS

Reverse-phase high-performance liquid chromatography (RP-HPLC) was used to quantify ALS (Shimadzu LC-20AT Pump, Standard Autosampler, SPD-20A UV/VIS Detector, Shimadzu, Kyoto, Japan). The polymeric phase was used in the chromatographic method for ALS analysis (Hamilton PRP-1) (5 µm pore size, 4.1 × 250 mm analytical column (Hamilton, Reno, NV, USA). The mobile phase was composed of a mixture of acetonitrile, methanol, and 0.05 M disodium hydrogenophosphate/0.05 M citrate trisodium (20:5:75), respectively [17]. The mixture of 0.05 M disodium hydrogenophosphate and 0.05 M citrate trisodium was prepared by adding 14.7 g/L of sodium citrate dihydrate and 7.05 g/ L of anhydrous dibasic sodium phosphate in water, and then adjusting with phosphoric acid to a pH of 8.0 before bringing the solution to volume. After degassing, the mobile phase was filtered through a 0.45 µm filter membrane. The flow rate of the mobile phase was set to 1 mL/min.

**Figure 2:**
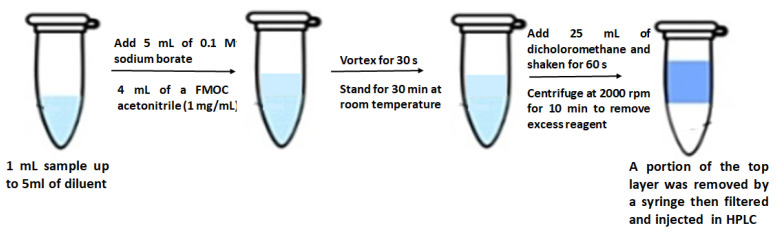
Schematic representation of sample preparation for quantification of ALS.

A derivatization reaction was performed for each sample in all experiments as shown in Figure 2. Here, 1 mL of each sample was collected and diluted to 5 mL by diluent in a 50 mL polypropylene centrifuge tube. After that, 5 mL of 0.1 M aqueous sodium borate solution was added to the previously diluted sample. Then, 4 mL of a 9-fluorenylmethyl chloroformate (FMOC) solution in acetonitrile with a concentration of 1 mg/mL was added. The tube was vortexed for 30 s and the reaction was allowed to stand for 30 min at room temperature. Subsequently, 25 mL of dichloromethane was added to the mixture and it was shaken for 30–60 s. The sample was then centrifuged at 2000 rpm for 10 min to remove excess reagent. Finally, a portion of the top layer (supernatant) was removed with a syringe and filtered before being injected into the HPLC [17].

For HPLC analysis, all injection volumes were 50 μL. At 266 nm, drug detection was performed. The limit of detection (LoD) and limit of quantification (LoQ) were calculated as defined by the International Conference on Harmonization guidelines (ICH) [18].

### 2.5. Statistical Analysis

A *t*-test analysis or one-way analysis of variance was used to statistically analyze the results when necessary (ANOVA). In each case, a statistically significant difference was defined as (*p* < 0.05). This was performed using the GraphPad Prism software (ver. 6; GraphPad, Inc., San Diego, CA, USA). 

## 3. Results and Discussion 

### 3.1. Characterization of ALS Loaded Niosomes

**Figure 3:**
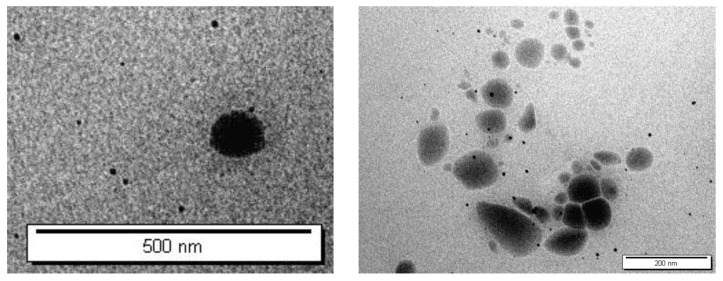
Transmission electron microscopy (TEM) micrographs of ALS-loaded niosomes (F4).

The stratum corneum (SC) in the outermost layer of the skin restricts the skin penetration of hydrophilic and macromolecular drugs [19]. To solve this problem, a number of strategies were used to improve transdermal drug delivery and to broaden the number of drugs delivered transdermally [6]. Therefore, various formulations of ALS-loaded niosomes were successfully prepared using the ether injection method. The bilayer vesicles were produced using different molar ratios of nonionic surfactant (Span 60, Tween 60, and Tween 80) and Chol. As a negative charged inducing agent, DCP was also used. The TEM micrographs of the niosomes are given in Figure 3. The TEM images confirmed the formation of niosomes. Indeed, TEM was employed to characterize niosomes in terms of shape, which illustrates that niosomes were of a spherical shape. 

The PS of the niosomal formulations was in the range of 99.6 ± 0.9 to 464.3 ± 6.1 nm, as summarized in Table 3. Particle size is an essential characteristic of drug delivery systems, affecting loading and release rates [20]. Skin deposition was not observed in studies where the particle size of carriers was greater than 600 nm [21]. A size of less than 300 nm may lead to an excessive amount of transdermal drug transport, whereas smaller carriers, such as those with a 300 nm particle size, enhance dermal delivery [22]. All formulations with a PS of more than 350 nm were excluded. This is because the approximate PS of vesicles to be able to deliver their contents into the deeper skin layers is 300 nm or below [23]. The hydrophilic–lipophilic balance (HLB) value is a critical factor in the formulation of niosomes. It has been noted that for higher niosome encapsulation efficiency, a HLB value between 4 and 8 is strongly advised [24]. Tween 60 is a surfactant with a high hydrophilicity (HLB = 14.9), whereas Span 60 is a nonionic surfactant with a hydrophobic portion (HLB = 4.7) and limited water solubility [25]. By combining Tween 60 with Span 60, the PS of the prepared niosome was larger than those containing Span 60 alone. Basiri, Rajabzadeh et al. (2017) observed that the lower hydrophilicity and bigger critical packing parameter (CPP) of Span 60 versus Tween 60 led into an increase in the average volume sizes [20]. According to the results, the size of niosomes showed a regular increase with an increase in the surfactant HLB values. An increase in the HLB value of the surfactant mixture and tighter packing of molecules inside the niosome could be the cause of this. A previous study that showed a similar outcome found that the size of niosomes increased when 20% Tween 60 was added. This finding may be related to the slightly larger hydrophilic portion of the Tween 60 molecule compared to the Span 60 using cephalexin [25]. The mean PS of the niosomes is also influenced by the membrane composition. The formula of niosomes with high ratio of Chol shows a bigger size than others. This might be interpreted in context of the fact that Chol would be more inclined to increase the number of bilayers [26]. This outcome is consistent with a prior study which showed that an increase in Chol induced the size of vesicles loaded with ciprofloxacin to increase [27]. The presence of Chol was found to be significantly effective in increasing the niosomal size (*p* > 0.05) [20].

The PDI is a measure of a sample’s heterogeneity [28]. The PDI ranged from 0 to 1, where values near to zero suggesting homogeneous dispersion [29], and less than 0.5 indicating a monodispersed sample [21]. As shown in Table 3, PDI ranged from 0.005 ± 0.00 to 0.334 ± 0.021, indicating that the vesicles are homogenous in size [30]. Therefore, the low PDI values indicated that the niosomal suspension had a narrow size dispersion and was homogenous. The ZP of colloidal systems is one of the characteristics used to interpret their stability. The charged particles repel one another as the ZP increases, stabilizing the system against aggregation. Colloidal systems with a zeta potential of higher than +30 mV or lower than −30 mV are considered stable [31,32]. Here, the ZP values of all formulations were noted to be in the range of −27.6 ± 0.22 and −42.27 ± 2.25 mV, as shown in Table 3, which is an indication of a stable system due to the electrostatic repulsion between nanovesicles [33].

Vesicle size, surfactant type, and Chol concentration are the factors affecting the effectiveness of entrapment [34]. Unencapsulated drug separated from the niosomal solution using centrifugation. After this step the encapsulated drug can be released from niosomes by lysing of vesicles. By completely disrupting the vesicle with isopropanol, the amount of drug entrapped in niosomes is evaluated [35]. Based on Table 3, the highest EE% of ALS were (65.94 ± 13.13%), (70.7 ± 7.35%), and (54.66 ± 1.69%) from F4, F6, and F10, respectively. The results indicated that the EE% for niosomes prepared using the mixtures of Span 60 and Tween 60 with HLB value 6.8 were superior to those prepared using solely Span 60 or others. The results are in agreement with a previous study of quercetin, which reported that using a mixture of Tween 60 and Span 60 results in the highest EE% (78.9%) of quercetin. Therefore, larger head groups and longer alkyl chains in the structure can result in greater vesicles to entrap larger amounts of quercetin [36]. The HLB value of the surfactant was modified using a mixture of Tween 60 (HLB = 14.9) and Span 60 (HLB = 4.7) to obtain a HLB value around 6.8, in order to obtain greater entrapment efficiency [37]. Ghafelehbashi, Akbarzadeh et al. (2019) observed that the amount of encapsulated cephalexin enhanced with an increase in the HLB value, which was in agreement with our results [25].

Charge-inducing agents are known to stabilize niosomes by raising their zeta potential. Additionally, they increase niosome membrane permeability to water, resulting in the production of large niosomes [38]. Here, F4, F6, and F10 were also prepared without using DCP. The EE% significantly decreased (*p* > 0.05) when removing DCP from the niosomal formulations, as shown in Table 4. Indeed, DCP, as a negatively charged molecule, is usually used in the niosomal formulation to prevent aggregation, which increases the stability of the niosomal dispersion. When a charge-inducing substance is added to the niosome membrane, water is allowed to enter the bilayer and the gap between the bilayers is increased [39]. Therefore, the incorporation of DCP was found to enhance the EE% significantly. This is because double hydrocarbon chains in DCP contributed to a tighter packing of the bilayer membrane which increased the EE%. This result was similar to a previous study [40], in which they revealed the influence of a stabilizer on EE%. Moreover, the phosphate groups of DCP aligned next to the polar heads of Span 60 and Tween 60 [20]. 

### 3.2. Attenuated Total Reflectance—Fourier Transform Infrared (ATR—FTIR) Analysis 

**Figure 4:**
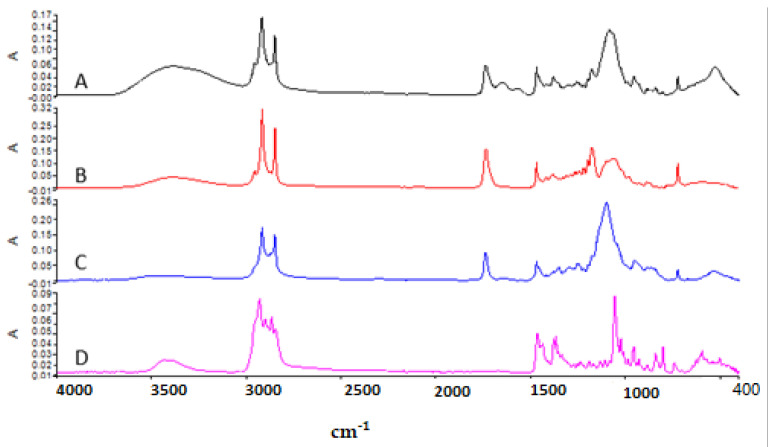
The FTIR spectrum of (**A**) blank niosome, (**B**) Span 60, (**C**) Tween 60, and (**D**) Chol.

In this study, ATR–FTIR analysis was used to evaluate functional groups found in the structure of ALS and to determine molecular interactions between niosome excipients and ALS. Figure 4 shows the ATR–FTIR of (A) blank niosome, (B) Span 60, (C) Tween 60, and (D) Chol. Span 60 showed the peaks around 3395 cm^−^¹ due to O–H stretching, at 2956 cm^−^¹ due to –CH stretching, and the strong C=O ester bond at 1736 cm^−^¹, which have also been reported in previous studies [41]. The ATR–FTIR spectrum of Tween 60 had a characteristic sharp peak around 1735 cm^−^¹ which is attributed to the stretching vibration of ester carbonyl [41]. Chol shows the wave number 3432 cm^−^¹ due to O–H stretching, 2930 cm^−^¹ due to C–H stretching, 1454 cm^−^¹ due to C=C stretching, and 1054 cm^−^¹ due to C–O bending vibrations [41]. The strong characteristic band in the region 3393 cm^−^¹ in blank niosomes is due to the existence of the O–H stretching vibration of the Chol, Span 60,and Tween 60 molecules [25].

**Figure 5:**
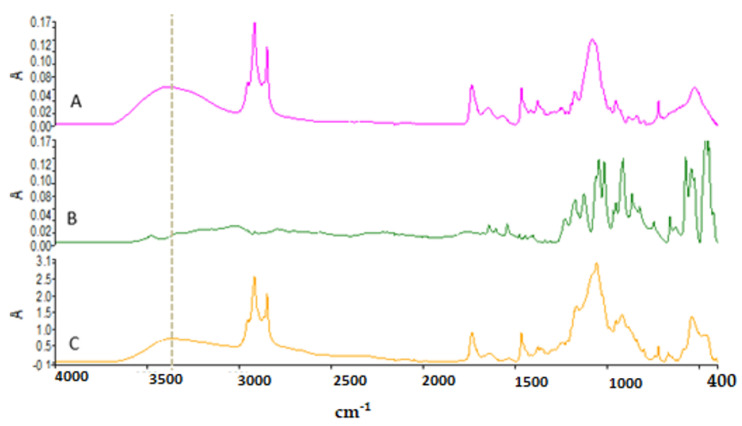
The FTIR spectrum of (**A**) blank niosome, (**B**) ALS (**C**), ALS-loaded niosome.

Figure 5 shows the ATR-FTIR of (A) Blank niosome, (B) ALS and (C) ALS-niosome. Major absorption peaks of ALS mainly appeared in wavenumber as follows; the ALS spectrum presented specific peaks on the region 1200–900 cm^−^¹ that correspond to C–O and P=O stretches, respectively [17]. The peak at 913 cm^−^¹ is due to hydroxyl group bending vibration, and the absorption peaks at 1016 cm^−^¹ and 1046 cm^−^¹ are due to P=O stretching vibrations, which are the characteristic peaks of ALS [42]. The characteristic peaks of the blank niosome was seen at 3393 cm^−^¹. This peak likely corresponds to O–H stretching of the ingredient between (Span 60, Tween 60, and Chol). In the ALS niosome, the OH– stretching peak was shifted to 3365 cm^−^¹, and this suggests the formation of H-bonds between ALS and the niosomes. Miladi et al. (2015) observed that the characteristic peak of ALS in the region 900 cm^−^¹ is also seen in chitosan nanoparticles loaded with ALS and absent in blank nanoparticles. These results confirmed that ALS is entrapped within niosomes [17]. 

In the spectrum of blank niosomes, the carbonyl dimer was observed to be shifted to 2918 cm^−1^, while the C=O stretching peak was observed to be shifted to 1737 cm^−1^. The shifts in the peaks corresponding to the carbonyl groups may be due to Span–Chol interactions, specifically hydrogen bonding, which is a characteristic of the formation of niosomes [43].

### 3.3. Stability Studies

The stability of niosome suspensions has always been a critical factor in the formulation process. A stable niosomal dispersion needs to have constant drug entrapment levels and particle sizes at storage conditions. Therefore, short-term stability testing of the highest drug entrapment efficiencies, namely F4, F6, and F10, was carried out for two months. The changes in PS, PDIs, ZP, and EE% during storage at 4 °C were summarized in Table 5. 

In all formulations over the period of the two months of storage, drug leakage was not noticed, since there was no significant difference in EE% (*p* > 0.05), as shown in Table 5. After two months, the EE% of ALS was 62.69 ± 3.01%, 67.27 ± 3.17%, and 52.22 ± 2.66% for K4, K6, and K10, respectively. The PS of the niosomes did not significantly (*p* > 0.05) change. Moreover, PDI values were found to be less than 0.1, which indicates the homogenous distribution and the stability of niosomes. In addition, there were no significant changes in the physicochemical parameters, such as appearance and color, and no precipitation was seen during the storage. Moreover, the ZP of all niosomes was within −35 mV, indicating a high formulation stability, and there were no significant changes (*p* > 0.05). The above results revealed that the F4, F6, and F10 formulations showed good physical stability at 4 °C after two months.

### 3.4. In Vitro Release Study 

**Figure 6:**
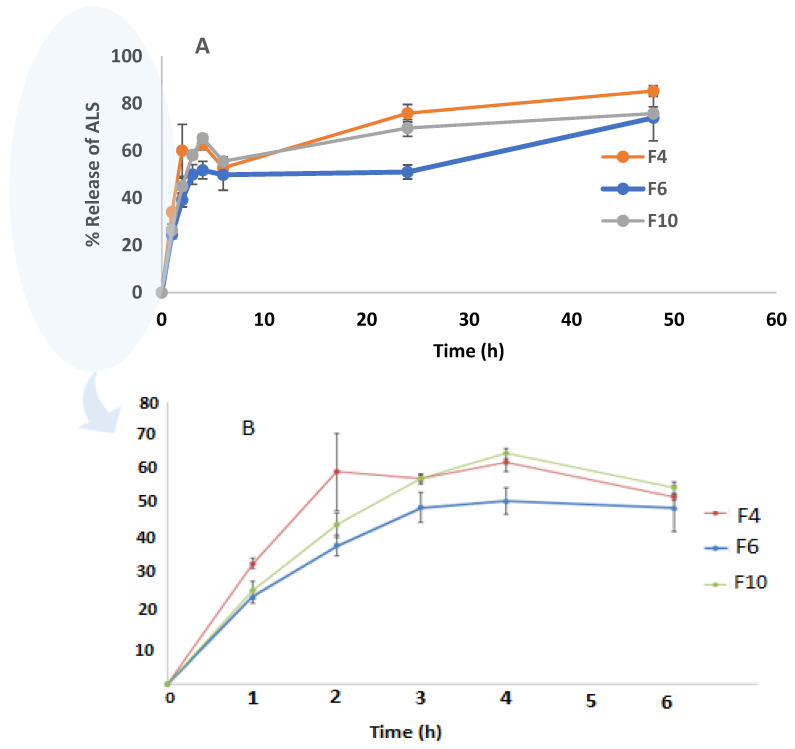
In vitro ALS release from F4, F6, and F10 niosomes for (**A**) 48 h and (**B**) 6 h (*n* = 3).

The drug release study was conducted for the highest EE% formulations (F4, F6, and F10) at up to 48 h, as shown in Figure 6. Drug release from the niosomes in F6 and F10 was slower than F4, which contained the lowest amount of Chol. Increasing the Chol content resulted in a decrease in the release rate. Shirvany, Rezayan et al. (2021) reported that Chol increases the strength of the membrane and reduces the release of cefazolin [44]. The release percentages of ALS from F4, F6, and F10 were 85.26 ± 2.3%, 73.91 ± 9.72%, and 76.00 ± 2.71%, respectively, after 48 h. There was no significant difference in the release of ALS between F4 and F6 (*p* = 0.120). Akbari et al. (2015) reported that there were no significant differences (*p* > 0.05) among the overall released amounts of ciprofloxacin (a hydrophilic drug) from the different surfactant type niosomes [45].

A rapid initial release that lasts for about 6 h was followed by a slower but continuous release period, resulting in the biphasic release of ALS from niosomes. This hydrophilic drug’s biphasic re-release behavior appears to be a characteristic of bilayer vesicles. Similar results were reported in the previous study for ciprofloxacin (a hydrophilic drug) niosomes which have a biphasic release [26]. The rapid initial phase may be attributed to drug desorption from niosome surface. Following the initial burst release, a continuous ALS release was seen for the next 48 h, which was caused by ALS diffusion from the lipid bilayer [46,47]. Based on the above release, F4 had the highest drug release out of all of the formulations. This is because the increase in the Chol amount lowered the release percentage from F6 and F10 niosomal formulations comparing with F4. Nishu, Karmoker et al. (2018) observed that Chol reduces the leakage or permeability of the encapsulating drug by decreasing the niosomal membrane fluidity of the soluble drug linagliptin, which may decrease release percentage [48].

### 3.5. MTT Cytotoxicity Assay

**Figure 7:**
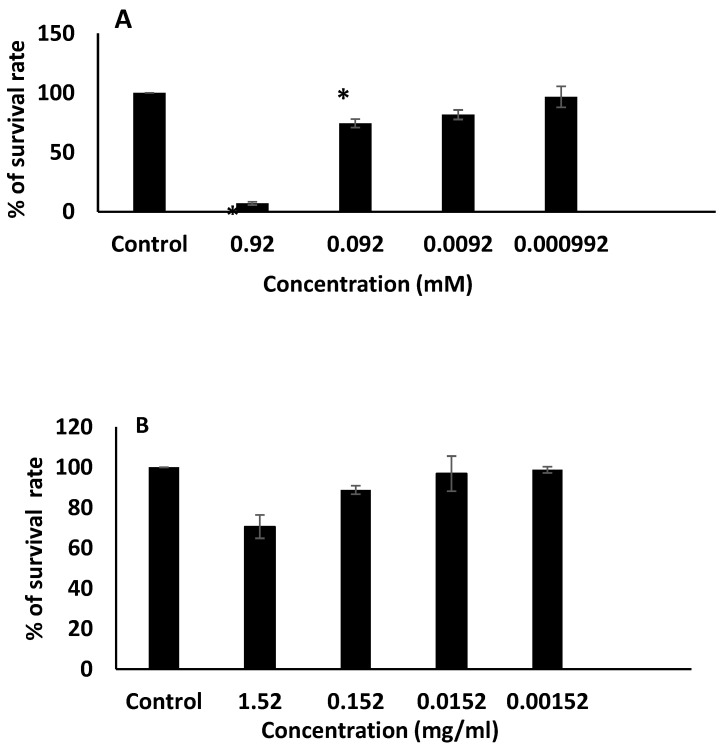
The cytotoxicity of (**A**) F4 and (**B**) blank F4 was evaluated by MTT assay. Each column represents the mean value ± SD (*n* = 3). * *p* < 0.01 indicate significant differences compared to the control group.

The cytotoxicity of empty niosomes and the ALS-loaded niosomes were assessed on a MCF-7 cell line using MTT assay. The MTT assay method is a sensitive technique for fast analysis of cell metabolic activity upon cell exposure to various biological molecules (Ganjooei et al., 2021). The viability of the breast MCF-7 cell was studied in the presence of F4 and blank F4. The effect of the formulations on the cell viability, as a function of concentration, is shown in Figure 7. We observed that drug-free niosomes had no significant toxicity on MCF-7 cells at the tested concentrations, (*p* > 0.01). Thus, blank F4 is biocompatible and nontoxic. Furthermore, F4 showed great inhibition of cell viability at 0.92 and 0.092 mM of ALS. The highest concentration of the ALS-loaded niosomes caused 95% inhibition of viability in MCF7 cells. The effect of ALS could be attributed to its inhibitory effect on bone resorption and bone-derived growth factors, leading to inhibition of tumor cell invasion, proliferation, and increased apoptosis in breast and prostate carcinoma [49]. Ilyas, Zarina et al. (2019) presented the first report on the cytotoxic potential of ALS on the HTB-breast cancer cell line [49].

### 3.6. Fabrication of Polymeric Microneedle Loaded ALS Niosomes

A wider range of drugs including hydrophilic drugs can now be administered through the skin due to advances in transdermal drug delivery, particularly with MNs [6]. Polymeric MNs have been powerful as a novel transdermal drug delivery platform for effective drug permeation, and have been widely used in the treatment of various diseases [50]. Polymeric MNs can eliminate sharp biohazard wastes and allow loading of non-potent drugs [51,52]. They can also facilitate appropriate therapeutic dosing by controlling the release kinetics of a pre-loaded drug [53]. 

Based on the characterization results of niosomes that have been obtained, F4 showed the highest percentage of drug entrapment and release percentage and, therefore, it was selected to be loaded into polymeric MN formulations. Those formulations showed the best physical characteristics upon removal from the mold, as they were hard but not brittle, and sharp, homogenous, and perfectly formed with an elegant appearance. Other formulations were neglected due to their poor physical characteristics upon removal from the mold, and some of them were very hard, brittle or swellable.

### 3.7. Morphology of MNs

It is common to use PVPs to manufacture polymeric MNs [54]. Studies showed that PVPs with low molecular weight could be completely eliminated by the kidney [54]. Therefore, PVP 40 KDa and PVA 14.5 KDa were chosen for fabrication of MNs. They are biocompatible polymers and their MW is less than 60 KDa; thus, they can be eliminated through the kidneys [55]. A 15 × 15 MN array of 250 μm × 600 μm (width and height), based on a pyramidal MN master template, was used to prepare MNs. Methylene blue was loaded into the MNs to facilitate observation. The resulting MNs measured approximately 595 µm in height and 250 µm in width at the base. A complete array of needles was formed, and the MNs baseplates were strong enough to be easily removed from the molds without causing damage to the array. The needles’ morphology was s square pyramidal shape. Due to their smaller aspect ratio, pyramidal MNs demonstrated greater mechanical strength than conical MNs, according to previous studies. [56].

### 3.8. Microneedles Dissolution in Skin

**Figure 8:**
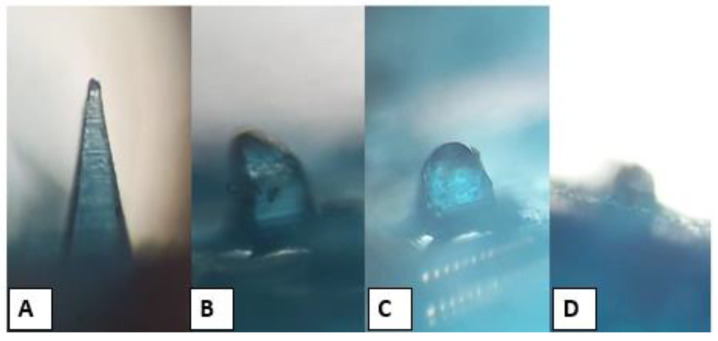
Morphological changes in the MN-3 after insertion into the rat skin at (**A**) 0, (**B**) 5, (**C**) 10, and (**D**) 20 min.

A polarizer microscope was used to investigate the changes in the shape of DMN formulations after 0, 5, 10, and 20 min following insertion into the skin. All formulations began to dissolve after insertion into the skin, and about 20% of the formulation was dissolved in the skin 5 min post-insertion. After 10 min, 50% of the needle length had dissolved, as shown in Figure 8. All formulations completely dissolved 20 min post-insertion, but MN-1 dissolved after 15 min post-insertion. It is also clearly observed that PVPs rapidly dissolve, and that the dissolving % increased as the PVP content increased [57]. Nguyen, Bozorg et al. (2018) reported that the use of PVA to fabricate doxorubicin MNs caused slow dissolution kinetics and that the rate of dissolution of MNs could be increased by using a combination of PVA and PVP in the polymer matrix [58].

### 3.9. Microneedles Insertion Studies

**Figure 9:**
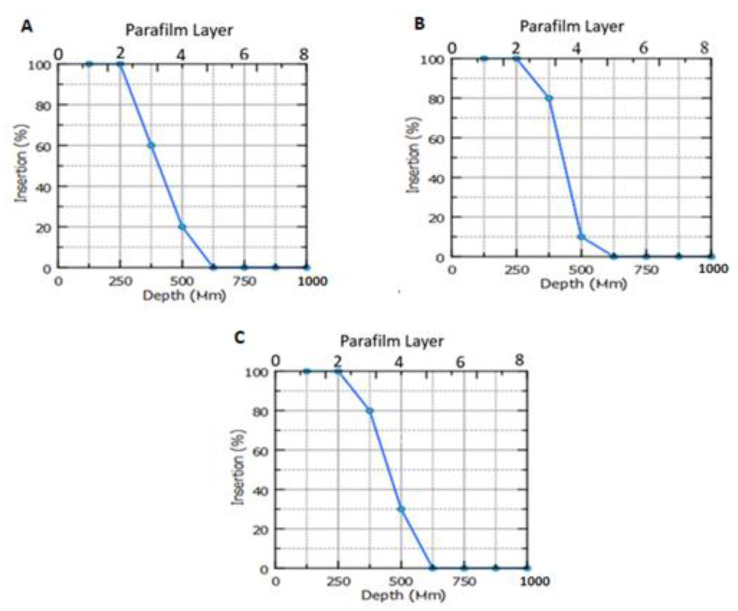
Parafilm™ insertion test for (**A**) MN-1, (**B**) MN-2, and (**C**) MN-3 following a manual force application (Means ± SD, *n* = 3).

As previously mentioned, Parafilm^TM^ was utilized as a validated artificial skin simulant for MN insertion investigations [14]. Figure 9 depicts the MNs’ insertion profile into the Parafilm^TM^. In this regard, the tested MN formulations did not fracture after applying the MNs manually. The insertion of most niosomal-loaded DMNs occurs between Parafilm^TM^ layer 2 and Parafilm^TM^ layer 3, which equates to distances between 252 and 378 µm. This is because each layer is approximately 126 µm thick [14]. In general, the mean thickness of the SC and epidermis of the skin is approximately 10–20 and 100–150 µm, respectively [19]. According to these findings, these MNs would be strong enough to pierce the SC and, subsequently, the upper dermis layer.

### 3.10. Determination of Drug Content in Niosomal DMNs

The drug content of niosomal DMNs from MN-1, MN-2, and MN-3 was evaluated and summarized in Table 6. The content uniformity test was carried out to see if the drug was distributed uniformly among different MNs. Percentage of drug recovery was calculated from various MNs for content uniformity within a single MN. The highest percentage recovery of ALS was from MN-1. However, there was no significant difference (*p* > 0.05) observed between percentage recoveries of ALS from all niosomal DMNs. 

### 3.11. Mechanical Characterization of Dissolving Microneedle Arrays

**Figure 10:**
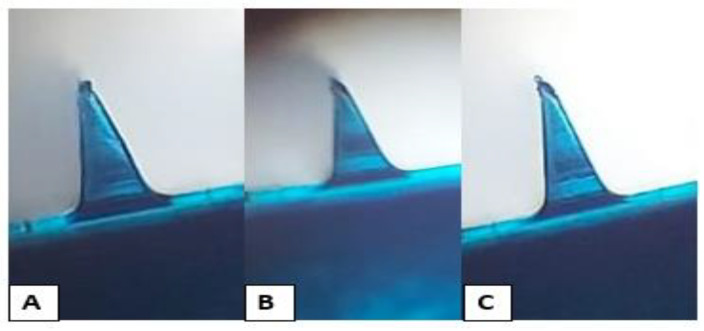
Polarizer photographs representing a decrease in the height of MN-3 after applying different forces of (**A**) 200 g, (**B**) 500 g, and (**C**) 1000 g.

The capability of MNs to be successfully inserted into the skin is critical, as the SC must be pierced for the MN to give its effect [59]. The changes of DMNs after applying different forces for 5 min are shown in Figure 10. By using the static force method, the weights that were applied ranged from 50 to 1000 g (Table 7), which is equivalent to 0.5 to 10 N, respectively.

The minimal fracture force of MNs that penetrated the skin without cracking was previously reported to be 0.058 N [60]. According to our findings, DMN formulations might potentially puncture skin because their fracture force was more than 0.058 N. The fracture force of niosomal MNs was obviously higher than reported fracture force. This is because the tips bended rather than breaking at higher forces. Consequently, the results suggested that niosomal MNs could penetrate through SC without fracture. The DMNs were visualized after testing using a polarizer microscope and by measuring the length of the MNs, as shown in Figure 10.

### 3.12. Stability Study 

The moisture content of MNs did not differ significantly (*p* > 0.05) in all formulations after one and two months, as shown in Table 8. Thus, niosomal MNs are considered stable within two months in term of moisture content. The percentages of drug recovery from MNs after one and two months were also not significantly different (*p* > 0.05). The recovery percentages of ALS after two months were 94.48 ± 4.80%, 93.09 ± 4.66%, and 90.11 ± 6.17%, for MN-1, MN-2, and MN-3, respectively. These results indicated the stability of ALS niosomes in all MN formulations.

### 3.13. Drug Permeation Study

**Figure 11:**
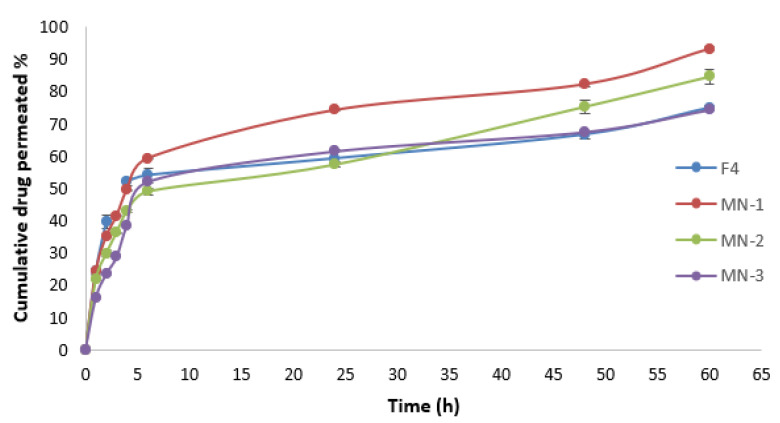
The cumulative amount (%) of ALS permeated through rat skin versus time (t) for MN-1, MN-2, MN-3, and F4 for 60 h (mean± SD, *n* = 3).

Figure 11 depicts the ALS permeation profile across rat skin after the application of niosome-loaded MNs and ALS niosomes as a control. It was found that the MN-1 delivered 1366.66 µg of ALS over a 60 h period, which equates to 93.44% of ALS being delivered. Meanwhile, the MN-2 delivered 1192.43 µg over a 60 h period, which equates to 81.53% of ALS being delivered, and MN-3 delivered 1108.77 µg over a 60 h period. This equates to 75.81% of ALS being delivered. The cumulative amount of ALS (Q) for different formulations was in the following order: MN-1 (containing 30% PVP) > MN-2 (containing 30% PVP:15% PVA (2:1)) > MN-3 (containing 30% PVP:15% PVA (1:1)), as seen in Table 9. These results may be attributed to the fact that PVP improves the solubility of PVA patches within the skin. Putri, Utami et al. reported that the greater the concentration of PVA, the slower the permeation of the ceftriaxone from DMN with a mixture of 40% PVP and 15% PVA [61].

The Jss of ALS was 62.18 ± 1.74, 53.64 ± 2.75, and 45.90 ± 4.05 µg/cm²/h for MN-1, MN-2, and MN-3, respectively. The Jss for different formulations was in the following order: MN-1 > MN-2 > MN-3, as summarized in Table 9. There was a significant difference (*p* < 0.05) in Jss between them. The findings demonstrated that an increase in PVA proportion reduces the drug release and enables sustained drug release, probably because considerably slower dissolution kinetics are involved [62], which is in agreement with a Lee, He et al. (2015) study which reported that using a mixture of PVP and PVA for fabrication of MNs can provide a sustained release [57].

Ex vivo skin permeation experiments revealed that the cumulative ALS permeated percentage observed using MN loaded niosomes (passive and active method) was better than the cumulative permeated percentage of ALS that permeated through F4 niosomes (passive method); the cumulative permeated percentages of ALS from F4 and MN-1 were 74.88 ± 0.79% and 93.14 ± 0.49%, respectively. The biggest difficulty with transdermal drug delivery is SC. Numerous strategies have been developed in order to penetrate the skin’s primary barrier. Due to its adaptability and capacity for sustained release, the integration of MNs with nano-systems has become more popular during the past 20 years [63,64]. Therefore, integrating physical and chemical technology provides a significant improvement in drug delivery. The use of MNs in combination with niosomes is the best possible approach to enhance the permeability and sustain the release of hydrophilic drugs [65]. This is because the encapsulation ALS in niosomes could increase the concentration of ALS by helping the niosomes to decrease transepidermal water loss, which improves SC hydration and loosens the stratum corneum’s closely packed cellular structure. In addition, niosome adsorption and/or fusion on the skin’s surface can result in a high drug thermodynamic activity gradient at the interface, which is the driving factor for drug permeation to be attached to the SC [66]. Additionally, MN-created micropores offer extra routes for niosome delivery to the dermis layer of the skin after the disruption of the SC barrier, making more of them available for systemic absorption through dermal microcirculation. Several previous studies were found in the literature describing MN-assisted permeation of nanoparticles [67,68]. Without utilizing any specific procedures, a new niosomally encapsulated DMN was produced in simple conditions at ambient temperature. The dual-delivery strategy utilizing niosomes, and MNs can enhance TDD while fostering the prolonged release of therapeutic substances [6]. The synergistic improvement in skin permeation caused by the combination of MNs and niosomes was explored for the first time in this study.

### 3.14. Drug Release Kinetics

**Figure 12:**
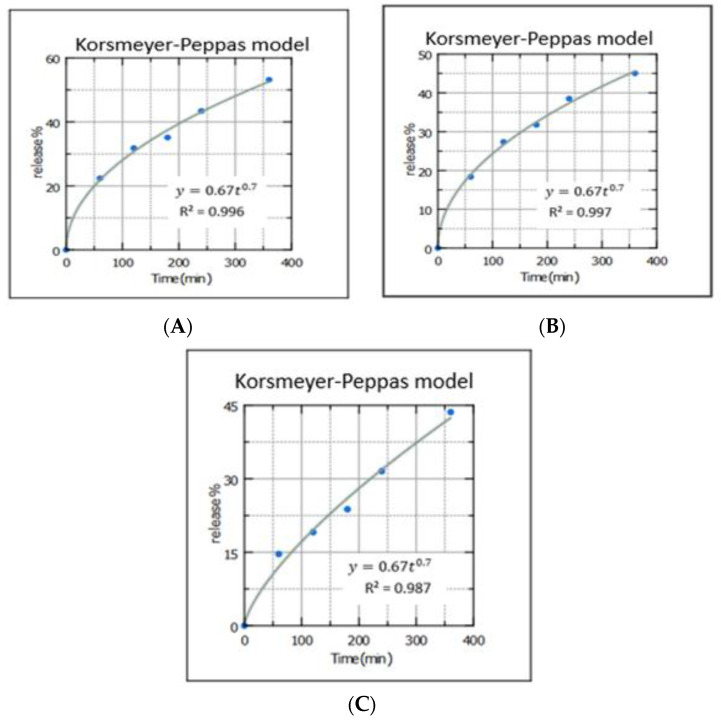
A mathematical model (Korsmeyer–Peppas) was applied to determine the kinetics governing the release of ALS through (**A**) MN-1, (**B**) MN-2, and (**C**) MN-3.

From Figure 12, the drug release of ALS from all formulations perfectly followed the Korsmeyer–Peppas release model as the drug release profile of ALS is closest to the trend line or regression line, and the highest values of the coefficient of correlation R² = 0.996, 0.997, and 0.987, respectively. To understand the mechanisms of release from MN-1, MN-2, and MN-3, it must be understood that in the Korsmeyer–Peppas model, the value of n describes the release mechanism of drug. 

The slope of the plot was constructed which described that the release exponent n was found to be 0.49, 0.49, and 0.7 for MN-1, MN-2, and MN-3, respectively, which implies that the drug release from MN-1 and MN-2 follows Fickian diffusion [69], while MN-3, with a high concentration of PVA, follows anomalous transport (non-Fickian diffusion). This implies that the release mechanism was governed by both diffusion and relaxation or erosion [70].

## 4. Conclusions

In an effort to minimize GI side effects and improve patient compliance, an alternative formulation for oral administration of ALS was investigated. A novel DMN was fabricated and loaded with niosomal formulation containing ALS under simple conditions at room temperature. An alternative administration route would make a significant contribution to patients. The dual-delivery approach of combining niosomes and MNs can improve TDD while promoting the sustained release of drug. In conclusion, a transdermal delivery of an ALS niosome loaded in DMNs was successfully prepared to provide sustained release of ALS for 60 h. 

## Figures and Tables

**Table 1:** The composition of ALS-loaded niosomes formulations.

HLB Value	DCP	Chol	Tween 80	Tween 60	Span 60	Code
	(mg)	(mg)	(mg)	(mg)	(mg)	
4.7	2	50	-	-	100	F1
4.7	2	100	-	-	100	F2
4.7	2	100	-	-	200	F3
6.8	2	50	-	20	80	F4
6.8	2	100	-	20	80	F5
6.8	2	100	-	40	160	F6
6.8	2	50	35	-	65	F7
6.8	2	100	35	-	65	F8
6.8	2	100	70	-	130	F9
6.8	2	100	-	16	64	F10
6.8	2	50	-	16	64	F11
6.8	2	100	-	32	128	F12

**Table 2:** Composition of ALS-loaded niosomes in MNs using different polymers PVP and PVA.

Code	ALS Niosomes	30%(*v*/*v*) PVP 40 kDa	15%(*v*/*v*) PVA 10 kDa	Ratio of PVP:PVA
MN-1	1.5 mL niosomes	2.50	0.00	1:0
MN-2	1.5 mL niosomes	1.66	0.84	2:1
MN-3	1.5 mL niosomes	1.25	1.25	1:1

**Table 3:** The PS, PDI, ZP, and EE% of prepared niosomal formulation. Results are represented by mean  ±  SD (*n*  =  3).

Code	PS (nm)	PDI	ZP (mV)	EE%
F1	136.00 ± 34.70	0.01 ± 0.00	−26.43 ± 0.85	17.95 ± 3.12
F2	193.60 ± 1.20	0.06 ± 0.02	−27.60 ± 0.22	27.76 ± 9.32
F3	172.00 ± 1.90	0.03 ± 0.03	−26.37 ± 2.62	25.04 ± 13.03
F4	269.60 ± 22.20	0.03 ± 0.02	−40.10 ± 4.07	65.19 ± 2.84
F5	303.60 ± 9.70	0.33 ± 0.02	−28.53 ± 1.83	43.37 ± 11.48
F6	319.30 ± 13.00	0.01 ± 0.00	−42.27 ± 2.25	74.71 ± 3.10
F7	382.80 ± 7.50	0.01 ± 0.00	−29.23 ± 1.81	33.37 ± 11.48
F8	449.60 ± 17.60	0.01 ± 0.00	−29.23 ± 2.37	23.32 ± 0.70
F9	464.30 ± 6.10	0.06 ± 0.02	−28.43 ± 2.00	28.06 ± 1.09
F10	229.50 ± 27.50	0.01 ± 0.12	−30.70 ± 2.71	54.27 ± 3.46
F11	99.60 ± 0.90	0.03 ± 0.00	−32.73 ± 2.95	23.47 ± 0.56
F12	193.0 ± 34.70	0.01 ± 0.00	−33.80 ± 2.50	27.71 ± 2.97

**Table 4:** Comparison of EE% of niosomal formulations (F4, F6 and F10) with and without DCP. Results are represented by mean  ±  SD (*n*  =  3).

Code	EE%	
	without DCP	with DCP
F4	30.24 ± 5.98	65.19 ± 2.84
F6	39.34 ± 16.93	74.71 ± 3.10
F10	28.18 ± 9.11	54.27 ± 3.46

**Table 5:** Short-term stability study results for F4, F6, and F10 at 4 °C after one and two months. Results are represented by mean  ±  SD (*n*  =  3).

Formula		PS	PDI	ZP	EE%
		(nm)		(mV)	
F4	One month	272.00 ± 17.40	0.04 ± 0.00	−36.13 ± 0.74	63.71 ± 1.54
	Two months	280.00 ± 22.70	0.05 ± 0.00	−34.35 ± 4.53	62.69 ± 3.01
F6	One month	322.30 ± 12.70	0.01 ± 0.00	−39.9 ± 2.43	74.83 ± 1.13
	Two months	330.45 ± 30.10	0.01 ± 0.00	−36.9 ± 1.44	67.27 ± 3.17
F10	One month	236.40 ± 29.50	0.01 ± 0.00	−34.7 ± 0.37	52.58 ± 3.23
	Two months	253.81 ± 43.70	0.01 ± 0.00	−34.6 ± 1.65	52.22 ± 2.66

**Table 6:** The drug content of ALS from niosomal DMNs; MN-1, MN-2, and MN-3 (Mean ± SD, *n* = 3).

Formulation	Drug Content (mg)	% Recovery
MN-1	1.39 ± 0.05	95.35 ± 4.30
MN-2	1.36 ± 0.08	93.20 ± 6.10
MN-3	1.33 ± 0.06	91.17 ± 4.96

**Table 7:** The reduction in height (μm) of MN-1, MN-2, and MN-3, tested as a function of forces of 200, 500, and 1000 g per array (Mean ± SD, *n* = 3).

Forces Applied per Array				Code
1000 g	500 g	200 g	Control	
557.0 ± 2.1	567.0 ± 5.0	582.0 ± 1.7	595.0 ± 1.2	MN-1
561.0 ± 7.6	571.0 ± 6.1	583.0 ± 1.6	597.0 ± 1.7	MN-2
574.0 ± 4.3	582.0 ± 1.7	592.0 ± 0.8	596.0 ± 0.5	MN-3

**Table 8:** Moisture content percentage and recovery percentage for MN-1, MN-2, and MN-3 after one and two months (Mean ± SD, *n* = 3).

Formulation	Moisture Content %		Recovery %	
	One Month	Two Months	One Month	Two Months
MN-1	4.31 ± 0.19	5.00 ± 0.34	94.97 ± 3.96	94.48 ± 4.80
MN-2	3.06 ± 0.19	4.31 ± 0.19	92.50 ± 5.88	93.09 ± 4.66
MN-3	3.73 ± 0.18	4.27 ± 0.37	90.82 ± 2.01	90.11 ± 6.17

**Table 9:** Drug permeation parameters (Jss and P) from different DMN formulations (mean ± SD, *n* = 3).

Code	Jss (µg/cm²/h)	P × 10^−2^ (cm/h)
MN-1	62.177 ± 1.73	4.36 ± 0.12
MN-2	53.636 ± 2.75	3.75 ± 0.01
MN-3	45.900 ± 4.04	3.22 ± 0.28

## Data Availability

Not applicable.
